# Molecular Epidemiology of Ascariasis: A Global Perspective on the Transmission Dynamics of *Ascaris* in People and Pigs

**DOI:** 10.1093/infdis/jiu193

**Published:** 2014-03-31

**Authors:** Martha Betson, Peter Nejsum, Richard P. Bendall, Rinki M. Deb, J. Russell Stothard

**Affiliations:** 1 Department of Production and Population Health, Royal Veterinary College, Hatfield, Hertfordshire, United Kingdom; 2 Department of Veterinary Disease Biology, Faculty of Health and Medical Sciences, University of Copenhagen, Denmark; 3 Department of Clinical Microbiology, Royal Cornwall Hospital, Truro; 4 Department of Parasitology, Pembroke Place, Liverpool School of Tropical Medicine, United Kingdom

**Keywords:** *Ascaris*, giant roundworm, population genetics, soil-transmitted helminth, zoonosis, neglected tropical disease, microsatellite, barcode

## Abstract

***Background.*** The roundworm *Ascaris lumbricoides* infects 0.8 billion people worldwide, and *Ascaris suum* infects innumerable pigs across the globe. The extent of natural cross-transmission of *Ascaris* between pig and human hosts in different geographical settings is unknown, warranting investigation.

***Methods.*** Adult *Ascaris* organisms were obtained from humans and pigs in Europe, Africa, Asia, and Latin America. Barcodes were assigned to 536 parasites on the basis of sequence analysis of the mitochondrial cytochrome c oxidase I gene. Genotyping of 410 worms was also conducted using a panel of microsatellite markers. Phylogenetic, population genetic, and Bayesian assignment methods were used for analysis.

***Results.*** There was marked genetic segregation between worms originating from human hosts and those originating from pig hosts. However, human *Ascaris* infections in Europe were of pig origin, and there was evidence of cross-transmission between humans and pigs in Africa. Significant genetic differentiation exists between parasite populations from different countries, villages, and hosts.

***Conclusions.*** In conducting an analysis of variation within *Ascaris* populations from pig and human hosts across the globe, we demonstrate that cross-transmission takes place in developing and developed countries, contingent upon epidemiological potential and local phylogeography. Our results provide novel insights into the transmission dynamics and speciation of *Ascaris* worms from humans and pigs that are of importance for control programs.

Ascariasis is caused by infection with the giant roundworm *Ascaris lumbricoides*, with around 760 million cases worldwide [1]. Although infections are particularly common in developing countries where sanitation and hygiene is poor, ascariasis exhibits a cosmopolitan distribution, with cases also described in developed countries [2–4]. It was recently estimated that ascariasis contributes 1.31 million disability-adjusted life years to the global burden of disease [1, 5]. The closely related parasite *Ascaris suum* infects innumerable pigs across the globe and is especially common in organic and extensive farming systems [6, 7]. Infections in pigs are associated with production losses owing to reduced growth and low feed conversion efficiency, with livers unfit for human consumption [7].

Because adult *A. lumbricoides* and *A. suum* worms are morphologically indistinguishable, there has been much debate as to whether they represent the same or different species [8, 9]. In addition, the extent of natural cross-transmission of worms between pig and human hosts is unclear [10]. Experimental cross-infections have demonstrated that *A. suum* can infect humans and that *A. lumbricoides* can infect pigs, with host preference in the efficiency of infection establishment [10, 11]. Although no definitive molecular marker has been found that can clearly distinguish between so-called human worms and pig worms, a combination of markers has proven useful [11]. Molecular studies have demonstrated that human *Ascaris* infections in developed countries are predominantly of pig origin [2–4, 12], whereas in developing countries human-to-human transmission predominates [13–16]. However, in China, 14% of human worms were derived from pigs (ie, were of zoonotic origin), indicating that cross-transmission in areas of endemicity may be more common than originally thought [17]. Hybrids between human and pig worms have been detected, indicating that mating can take place between *Ascaris* from the 2 host species [18].

Molecular markers have been used to investigate geographical differences in the population structure of *Ascaris* on a macroscale and microscale. Overall, the data support structuring of *Ascaris* populations between countries [13, 18–21]. However, results were more variable at a local scale [14, 19, 20, 22], which may reflect methodological differences and variations in transmission dynamics, migration, and farming practices. To date, sampling of *Ascaris* from Africa has not been extensive. Given that humans arose on this continent [23], it is plausible that there has been a long association between humans and the *Ascaris* parasite here.

In this study, we used mitochondrial and microsatellite markers to characterize a large collection of adult *Ascaris* worms acquired from human and pig hosts in areas where *A. lumbricoides* is considered endemic or nonendemic. The level of *Ascaris* cross-transmission between pig and human hosts was investigated, as was the geographical structuring of parasite populations. The results provide novel insights into the evolutionary origins of *Ascaris* in humans and pigs.

## MATERIALS AND METHODS

### *Ascaris* Samples, Ethics Approval, and Informed Consent

The host and sampling location of the worms are summarized in Table 1. Many samples were collected during previous studies. Additional worms were obtained from humans in the United Kingdom, Kenya, and Bangladesh and from pigs in the United Kingdom, Denmark, and Tanzania. Ethics approval and informed consent were obtained for collection of worms from humans [13, 14, 19, 20, 24, 26]. Sixteen worms from humans had been submitted to the Clinical Microbiology Laboratory, Royal Cornwall Hospital, for routine identification.

**Table 1.** Characteristics of *Ascaris* Samples Included in This Study

Country, Location	Host	Samples, No.	*cox*1	MS	Reference
United Kingdom					
Cornwall	Humans	15	14	11	[3], this study
Abattoir, Bedfordshire	Pigs	49	38	41	This study
Denmark					
Unspecified	Humans	2	2	2	This study
Unspecified	Pigs	31	27	31	This study
Uganda					
Mayengo, Kabale District	Humans	10	10	10	[24]
Nyamirima, Kabale	Humans	9	9	9	[24]
Katuna, Kabale	Humans	1	1	1	[24]
Kiniogo, Kabale	Humans	9	9	8	[24]
Nyakitokoli, Kabale	Humans	41	41	18	[24]
Habutobere, Kabale	Humans	37	37^a^	32^a^	[20]
Musezero, Kisoro District	Humans	62	61^a^	56^a^	[20]
Hamukaaka, Kabale	Humans	19	19^b^	9^b^	[19]
Kapchorwa, Kapchorwa District	Humans	2	2	0	Davies et al (unpublished data)
Katuna, Kabale	Pigs	23	23	21	[25]
Burambira-Katuna, Kabale	Pigs	6	6	5	[25]
Kamugangguzi, Kabale	Pigs	11	11	9	[25]
Rwakakobe, Kabale	Pigs	12	8	9	[25]
Mugyenyi Lane, Kabale	Pigs	7	7	6	[25]
Abattoir, Kampala	Pigs	55	52	6	This study
Zanzibar					
Kandwi	Humans	5	5^b^	4^b^	[19]
Kizimibani	Humans	8	8^b^	8^b^	[19]
Ghana	Humans	18	15^b^	16^b^	[19]
Tumbatu-Jongowe	Humans	29	29^b^	25^b^	[19]
Miscellaneous	Humans	2	2^b^	0	[19]
Tanzania					
Abattoir	Pigs	38	37	36	This study
Kenya					
Kwale	Humans	6	6	0	This study
Zambia					
Traveler (worm sampled in the United Kingdom)	Humans	1	1	0	This study
Bangladesh					
Unspecified	Humans	41	37	37	This study
Nepal					
Unspecified	Humans	5	5	0	[21]
Philippines					
Unspecified	Pigs	6	3	0	[13]
Guatemala					
Santa Cruz Naranjo and Chaimal, Santa Rosa Province	Humans	6	6	0	[14, 26]
Santa Cruz Naranjo, Santa Rosa	Pigs	6	5	0	[14, 26]
Total	Humans and pigs	572	536	410	…

Abbreviation: MS, microsatellite.

^a^ *Cox*1 and microsatellite data published [20].

^b^ *Cox*1 and microsatellite data published but only 5 microsatellite loci were analysed in the published study [19].

### DNA Extraction

Genomic DNA was extracted from worm gonads or muscle tissue [4, 19], using the CTAB method [27], the Genomic DNA Isolation Kit for Tissue and Cells (nexttec, Hilgertshausen, Germany), or the DNeasy Blood and Tissue Kit (Qiagen, Manchester, United Kingdom).

### *Cox*1 Sequencing and Microsatellite Amplification

A 450-bp fragment of the *Ascaris cox*1 gene was amplified using primers As-Co1F and As-Co1R [15], and the fragments were sequenced [19]. Eight microsatellite loci (ALAC07, ALAC09, ALGA48, ALTN04, ALGA31, ALGA15, ALAC32, and ALAC08) were individually amplified by polymerase chain reaction from a subset of samples (Table 1), and fragment sizes were determined, as published elsewhere [28].

### Sequence Analysis

*cox*1 sequences were assembled and manually edited using Sequencher v4.8 (Gene Codes Corporation, Ann Arbor, MI). A 383-bp consensus sequence was obtained for each sample and aligned using MacClade v4.08 (Sinauer Associates, Sunderland, MA). Collapse v1.2 was used to identify samples with identical haplotypes. BLAST was used to search for exact sequence matches in GenBank and novel haplotypes were submitted to GenBank (accession numbers KF719094–151). A minimum-spanning parsimony network was constructed in TCS v2.1. jModeltest v2.1.3 was used to determine the best-fitting nucleotide substitution model [29], using a data set containing all unique haplotypes and a selection of previously identified haplotypes (CavHap1, CavHap3, CavHap5, and CavHap13–9) [12] obtained from GenBank. The resulting model (Hasegawa-Kishino-Yano, with gamma distributed rates) was used for construction of a maximum likelihood tree in MEGA v5.05, with branch support provided by bootstrapping (1000 replications). The same software was used to determine *p*-distances between clusters.

### Microsatellite Analysis

Microsatellite allele sizes were determined using PeakScanner v1.0 (Applied Biosystems). PowerMarker v3.25 [30] was used to determine the number of alleles per locus (N_A_), observed heterozygosity (H_O_), and expected heterozygosity (H_E_). Numbers of private alleles (N_P_) and allelic richness (R_A_) were calculated using FSTAT v2.9.3.2 [31]. To take into account potential relatedness of parasites from the same host individual, genetic differentiation between populations was analyzed using the hierarchical analysis of molecular variance procedure in Arlequin v3.5.1.2 [32]. Pairwise estimates of F_ST_ (a measure of genetic differentiation) between populations were generated, and permutation tests of genetic differentiation (101 000 permutations) were conducted. Genetic distances between parasite populations (Cavalli-Sforza and Edwards' chord distances) [33] were estimated in PowerMarker and visualized using a neighbor joining clustering algorithm with bootstrapping (1000 replications) to determine phenogram reliability. Consense (Phylip v3.65) was used to compute a consensus tree, which was visualized using DrawTree (Phylip v3.65). This was done for *Ascaris* populations from different host types, countries, villages, or individuals (in which the number of worms per village/host was ≥6).

Population structure was also inferred from the microsatellite data set using Bayesian analysis in STRUCTURE v2.3.4 [34, 35]. An admixture model assuming correlated allele frequencies was used (burn-in length, 50 000; run length, 100 000). Values of *K* from 1 to 14 were tested, and 20 independent runs were performed for each *K*. To determine the most likely true value of *K*, the mean Ln P(D) value (an estimate of posterior probability) was determined for each *K* and plotted against *K*. If there was no obvious peak in the plot, Δ*K* was calculated and plotted against *K* [36]. This analysis indicated that 2 was the most likely true value of *K*. To examine whether further substructuring existed, samples were assigned to one of 2 groups on the basis of a *Q* value of >0.5 in the STRUCTURE output. The simulation was run again for each group separately, testing values of *K* from 1 to 10, using a burn-in of 100 000 and a run length of 1 000 000 to improve stability between runs. The presence of cross-transmission and hybrids between pig and human worms was investigated using STRUCTURE, NewHybrids [37], and BAPS [38], as described elsewhere [17, 18].

## RESULTS

### *Cox*1 Haplotypes

We analyzed *cox*1 sequences from 319 *Ascaris* worms collected from humans and 217 worms collected from pigs (Table 1). A total of 75 different haplotypes were identified, 43 of which were novel. Table 2 summarizes haplotype abundance by host and location. H1 was by far the most abundant haplotype, particularly in worms from humans. H3 was also common in human *Ascaris*. In contrast, H7, H28, H52, and H64 were abundant in pig worms, but of these only H64 was unique to *Ascaris* from pigs.

**Table 2.** *Cox*1 Haplotype Abundance, by Country and Host

Haplotype	UK		DK		UG		ZZ	TZ	KE	ZA	BA	NP	PH	GT	
	H	P	H	P	H	P	H	P	H	H	H	H	P	H	P
H1	…	…	…	…	134	4	29	…	1	…	23	…	…	…	…
H2	…	…	…	…	2	…	1	…	…	…	…	…	…	…	…
H3	…	…	…	…	23	…	12	…	…	…	…	…	…	1	…
H4	…	…	…	…	…	…	3	…	…	…	…	…	…	…	…
H5	…	…	…	…	…	…	2	…	…	…	…	…	…	…	…
H7	…	5	…	4	1	33	2	1	…	…	…	…	…	2	…
H8	…	…	…	…	…	…	2	…	…	…	1	…	…	…	…
H11	…	…	…	…	2	…	1	…	…	…	1	…	…	…	…
H21	…	…	…	…	2	…	…	…	…	…	…	…	…	…	…
H24	…	…	…	…	1	…	…	…	…	…	1	…	…	…	…
H28	8	8	1	19	1	16	…	28	…	…	…	…	2	…	3
H29	…	…	…	…	2	…	…	…	…	…	…	…	…	…	…
H32	…	…	…	…	…	3	…	2	…	…	1	2	…	2	…
H33	…	…	…	…	…	1	…	…	…	…	1	…	…	…	…
H47	…	…	…	…	2	…	…	…	…	…	…	…	…	…	…
H52	1	…	…	…	…	41	…	…	…	…	…	…	…	…	…
H57	…	…	…	…	…	3	…	…	…	…	…	…	…	…	…
H60	…	…	…	…	…	…	…	…	…	…	…	…	…	…	2
H64	…	22	…	1	…	2	…	3	…	…	…	…	…	…	…
H66	…	1	…	…	…	…	…	1	…	…	…	…	…	…	…
H74	2	…	…	…	…	…	…	…	…	…	…	…	…	…	…
H75	…	…	…	…	…	…	…	…	4	1	…	…	…	…	…
H77	…	…	…	…	1	…	…	…	1	…	…	…	…	…	…
Rare^a^	3	2	1	3	18	4	7	2	…	…	9	3	1	1	0
Total	14	38	2	27	189	107	59	37	6	1	37	5	3	6	5

Abbreviations: BA, Bangladesh; DK, Denmark; GT, Guatemala; H, humans; KE, Kenya; NP, Nepal; P, pigs; PH, Philippines; TZ, Tanzania; UG, Uganda; UK, United Kingdom; ZA, Zambia; ZZ, Zanzibar.

^a^ Haplotypes that were only sampled once in this study.

**Figure 1.**
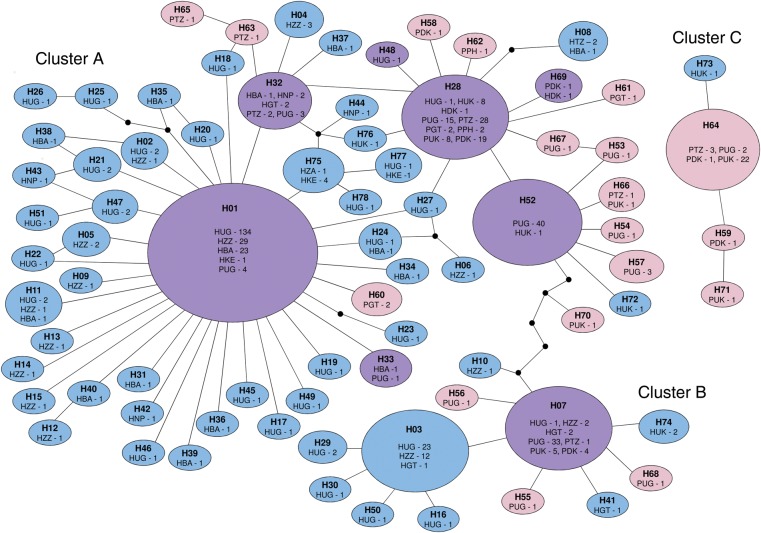
Minimum spanning TCS network of all *cox*1 haplotypes identified. A line indicates 1 base change. A black dot indicates a nonsampled or extinct haplotype. The size of the ovals is representative of the number of samples with a particular haplotype. Blue ovals represent haplotypes only found in worms from humans, pink ovals indicate haplotypes only found in worms from pigs, and purple ovals indicate haplotypes identified in worms from both hosts. The haplotype number is displayed in bold. The host type and geographical location are also indicated. Numbers indicate number of samples from each host type and location with the specific haplotype. The 3 clusters are labeled cluster A, cluster B, and cluster C. Abbreviations: BA, Bangladesh; DK, Denmark; GT, Guatemala; H, humans; KE, Kenya; NP, Nepal; P, pigs; PH, Philippines; TZ, Tanzania; UG, Uganda; UK, United Kingdom; ZA, Zambia; ZZ, Zanzibar.

In a minimum spanning parsimony network of all haplotypes, 3 main groups could be observed (Figure 1). Cluster C sequences differed by >8 base pairs from other haplotypes and so were not connected to the main network. There was no obvious segregation of haplotypes on the basis of geographical location. Clusters A and B contained haplotypes from both pig and human worms, but there was a tendency for haplotypes from human *Ascaris* to cluster around H1 and H3 and those from pig *Ascaris* to cluster around H7, H28, and H52. Cluster C only contained 1 sequence from a human worm (from the United Kingdom). A maximum likelihood tree was constructed on the basis of these haplotypes and included additional, recently published *Ascaris cox*1 sequences [12] (Supplementary Figure 1). The tree revealed the same 3 groupings of *Ascaris* sequences with strong bootstrap support for separation between the 3 groups. Mean *p*-distances were 2.8% between clusters A and B, 5.4% between clusters A and C, and 4.2% between clusters B and C. None of the sequences from other ascarids fell into these groups. Whereas clusters A and B contained sequences from *Ascaris* collected from both host species and all 4 continents, cluster C contained only sequences from Europe and Africa.

### Microsatellite Analysis

Microsatellite data were analyzed from 246 human and 164 pig worms (Table 1). Microsatellite allelic diversity stratified by locus, host species, and location is summarized in Supplementary Table 1. Mean allelic richness was somewhat higher in *Ascaris* from human hosts than those from pigs (*P* = .062; 15 000 permutations); this was more apparent when *Ascaris* from humans in the United Kingdom were classified as pig *Ascaris* (*P* = .041). There was no difference in observed or expected heterozygosity between human and pig *Ascaris* (H_O_, *P* = .426; H_E_, *P* = .200).

**Figure 2.**
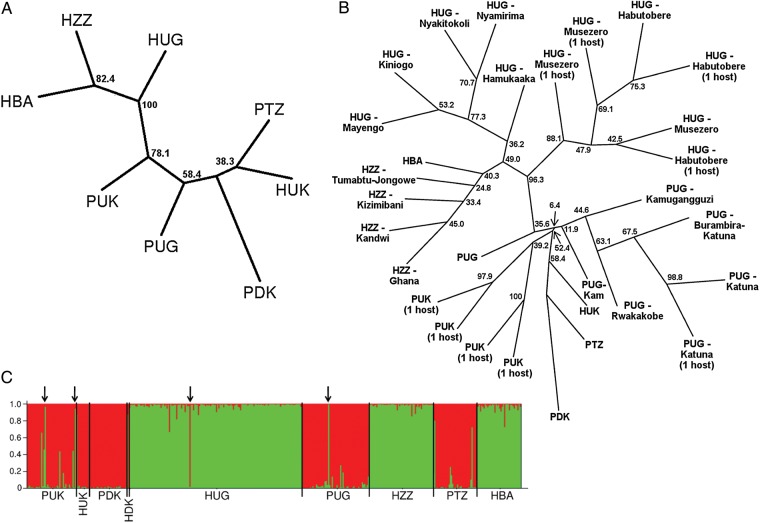
*A*–*B*, Cavalli-Sforza and Edwards' chord distances between *Ascaris* populations from different host types and locations represented in consensus neighbor joining trees based on 8 microsatellite markers. *A*, Populations stratified by host type and country. *B*, Populations stratified by host type and by country, by village, or by individual host, depending on the number of worms sampled for each location and host and whether information on individual hosts was available. Names of villages are provided in panel *B*, and if a population corresponds to worms from only 1 host, this is indicated in brackets. Bootstrap values are displayed and are based on 1001 replications. *C*, Assignment of *Ascaris* samples to clusters on the basis of STRUCTURE analysis. Output from a representative STRUCTURE run is presented. One cluster is indicated in red (mostly worms from pigs) and green (mostly worms from humans). Each narrow column corresponds to 1 sample. Examples of cross-transmission between human and pig hosts are indicated with black arrows. In addition, most worms from humans in the United Kingdom and Denmark appear to have originated from pigs. Abbreviations: BA, Bangladesh; DK, Denmark; GT, Guatemala; H, humans; KE, Kenya; NP, Nepal; P, pigs; PH, Philippines; TZ, Tanzania; UG, Uganda; UK, United Kingdom; ZA, Zambia; ZZ, Zanzibar.

Genetic flow between *Ascaris* populations from different hosts and locations was investigated through estimation of pairwise F_ST_ values and permutation tests of genetic differentiation (Table 3). There was evidence of genetic differentiation between nearly all *Ascaris* populations. However, differentiation between worms from sympatric pig and human hosts was higher than between populations from the same host in different geographical areas, except for human worms from United Kingdom. When genetic distances between *Ascaris* populations were visualized using a consensus neighbor-joining tree, populations from pig hosts clustered together and populations from humans grouped together, apart from United Kingdom human worms, which clustered with pig worms (Figure 2*A*). Populations were then subdivided on the basis of village or individual host, and genetic distances were determined (Figure 2*B*). The same major division between worms from pig and human hosts was observed, with further clustering by location. *Ascaris* from humans in Uganda divided into 2 main clusters, one containing worms from Habutobere and Musezero and the other containing worms from other villages. The worms from Bangladesh grouped with those from Zanzibar. *Ascaris* worms from humans in the United Kingdom appeared most similar to worms from pigs in Tanzania and Denmark. *Ascaris* populations from different pig hosts in the United Kingdom were genetically differentiated.

**Table 3.** Pairwise Differentiation Between *Ascaris* Populations From Different Hosts and Countries, Based on Microsatellite Data

Host, Country	Host, Country							
	P, UK	H, UK	P, DK	H, UG	P, UG	H, ZZ	P, TZ	H, BA
P, UK	…	.153	.030	.001	.005	.025	.059	.004
H, UK	.037	…	.001	<.0001	.0002	.122	.009	<.0001
P, DK	.057	.029	…	<.0001	.0002	.012	<.0001	<.0001
H, UG	.065	.092	.101	…	<.0001	.013	<.0001	<.0001
P, UG	.056	.041	.050	.100	…	.004	<.0001	<.0001
H, ZZ	.056	.050	.070	.033	.068	…	.069	<.177
P, TZ	.064	.040	.071	.116	.051	.071	…	<.0001
H, BA	.065	.081	.100	.051	.092	.012	.106	…

Data below the diagonal denote pairwise F_ST_ (a measure of genetic differentiation) values based on hierarchical analysis, and data above the diagonal denote *P* values from the permutation test of genetic differentiation (10 100 permutations).

Abbreviations: BA, Bangladesh; DK, Denmark; H, humans; P, pigs; TZ, Tanzania; UG, Uganda; UK, United Kingdom; ZZ, Zanzibar.

### Bayesian Analysis

Bayesian analysis of the microsatellite data, using STRUCTURE, was used to assign worms to clusters without prior knowledge of host type or sampling location. On the basis of posterior probabilities, the true value of *K* (ie, the number of clusters) was most likely 2. A bar plot from a representative STRUCTURE run is shown in Figure 2*C*. It was apparent that the 2 clusters corresponded to host type, apart from worms from humans in Europe, which mainly clustered with worms from pigs. To investigate further substructuring in the data set, worms were designated to one of 2 groups on the basis of the STRUCTURE output, and the simulation was run again for each group separately. Additional structure was evident, with a likely *K* of 4 for each group. The 4 clusters for human worms loosely corresponded to location (Table 4): Uganda (Musezero and Habutobere), Uganda (other villages), Zanzibar, and Bangladesh. However, clusters 3 and 4 both contained worms from Zanzibar and Bangladesh. For the group mainly consisting of pig worms (Table 4), the clusters also corresponded to geographical area: United Kingdom (cluster 1), Denmark (cluster 2), Uganda (cluster 3), and Tanzania (cluster 4). The human worms from Denmark and the United Kingdom shared genomes mainly with pig worms from Denmark (cluster 2), and the human worm from Uganda mainly with pig worms from Uganda (cluster 3).

**Table 4.** Proportions of Each *Ascaris* Population Originating Predominantly From Humans and Pigs That Were Assigned to Each of the 4 Clusters Identified in STRUCTURE

Population, Host and Country	Worms, No.	Cluster 1	Cluster 2	Cluster 3	Cluster 4
Human *Ascaris*					
P, UK	3	0.406	0.013	0.027	0.553
H, UK	1	0.178	0.005	0.006	0.811
H, DK	1	0.179	0.005	0.006	0.809
H, UG	142	0.568	0.326	0.047	0.059
P, UG	1	0.978	0.006	0.009	0.067
H, ZZ	53	0.017	0.113	0.741	0.129
P, TZ	2	0.110	0.035	0.065	0.790
H, BA	37	0.044	0.051	0.267	0.638
Pig *Ascaris*					
H, UG	1	0.021	0.073	0.559	0.347
P, UG	55	0.034	0.145	0.751	0.071
P, TZ	34	0.012	0.361	0.016	0.611
P, UK	38	0.708	0.160	0.035	0.096
H, UK	10	0.045	0.707	0.060	0.188
P, DK	31	0.038	0.843	0.083	0.037
H, DK	1	0.008	0.844	0.094	0.055

Data are proportion of worm genomes, unless otherwise indicated.

Abbreviations: BA, Bangladesh; DK, Denmark; H, humans; P, pigs; TZ, Tanzania; UG, Uganda; UK, United Kingdom; ZZ, Zanzibar.

To investigate whether there were any cases of cross-transmission and hybridization between worms from human hosts and worms of pig origin, simulations using BAPS, NewHybrids, and STRUCTURE were conducted (Table 5). Hybrids were identified by STRUCTURE but not by BAPS or NewHybrids, suggesting that these represented false positives. All programs identified 12 worms from humans in the United Kingdom and Denmark and 1 worm from humans in Uganda as belonging to the pig cluster. In addition, 2 *Ascaris* from pigs in the United Kingdom and 1 worm from Uganda were assigned to the human cluster. These worms likely represent examples of cross-transmission between humans and pigs (Supplementary Table 2).

**Table 5.** Summary of STRUCTURE, BAPS, and NewHybrids Analyses

Host, Country, Program	Population 1	Population 2	Hybrid	NC	Total
Human *Ascaris*					
United Kingdom and Denmark					
STRUCTURE	0	11	2	0	13
BAPS	0	13	0	0	13
NewHybrids	2	11	0	0	13
Uganda					
STRUCTURE	140	1	2	0	143
BAPS	142	1	0	0	143
NewHybrids	140	3	0	0	143
Zanzibar					
STRUCTURE	54	0	0	0	53
BAPS	54	0	0	0	53
NewHybrids	53	1	0	0	53
Bangladesh					
STRUCTURE	37	0	0	0	37
BAPS	37	0	0	0	37
NewHybrids	36	0	0	1	37
Pig *Ascaris*					
United Kingdom					
STRUCTURE	2	34	5	0	41
BAPS	4	37	0	0	41
NewHybrids	5	35	0	1	41
Denmark					
STRUCTURE	0	31	0	0	31
BAPS	0	31	0	0	31
NewHybrids	0	31	0	0	31
Uganda					
STRUCTURE	1	52	3	0	56
BAPS	1	55	0	0	56
NewHybrids	1	54	0	1	56
Tanzania					
STRUCTURE	0	33	3	0	36
BAPS	2	34	0	0	36
NewHybrids	2	34	0	0	36

Data are no. of worms.

Abbreviation: NC, not classified.

## DISCUSSION

Here, we describe the first large-scale molecular analysis of human and pig *Ascaris* from different locations across the globe using mitochondrial and microsatellite markers including better sampling of African isolates. We found strong genetic differentiation between *Ascaris* originating from human and pig hosts. However, there was also evidence of cross-transmission of *Ascaris* between pigs and humans, with practically all European human *Ascaris* infections originating from pigs and sporadic zoonotic and anthroponotic transmission in areas of endemicity.

The *cox*1 haplotypes identified add to the growing database of *Ascaris cox*1 sequences from human and pig hosts [12, 15, 19, 20, 39, 40]. They fell into 3 groups, which correspond to clusters A*–*C, identified previously [12, 40]. The *p*-distances between cluster C and clusters B and A were surprisingly high but still lower than the 10% that is normally seen for *cox*1 sequences between different helminth species [41]. In addition, it should be noted that the existence of 3 taxonomic clusters was not supported by the microsatellite data, highlighting the complex nature of mitochondrial inheritance and the pitfalls associated with using a single marker for molecular epidemiological studies [42]. The extremely frequent sampling of H1 and its central position in cluster A suggest that this may be an ancestral haplotype, originating in Africa, that has subsequently diverged and spread. Moreover, we did not find a strict segregation between *cox*1 haplotypes from worms of human or pig origin. Nevertheless, there were constellations of *Ascaris* haplotypes originating predominantly from 1 host species, suggesting that there are local barriers to the exchange of genetic material between pig and human *Ascaris* and adaptations to particular host species. Interestingly, haplotypes from human *Ascaris* in the United Kingdom and Denmark cluster practically exclusively with pig worms, indicating a zoonotic origin for these infections.

Using different approaches for analysis of microsatellite data, we observed genetic differentiation between human and pig *Ascaris* in developing countries. These results are in accordance with findings from a number of published studies [4, 13, 16–18, 43]. In contrast, *Ascaris* from humans in Europe clustered with pig worms rather than human worms from other areas, providing further evidence that *Ascaris* infection is a zoonosis in developed countries [2–4, 12]. Using Bayesian analysis, there were no worms of pig origin found in humans from Zanzibar or Bangladesh, which is unsurprising given that both are Muslim countries where pig farming is uncommon. In Uganda, where around 18% of households keep pigs [44], 1 of 143 human worms (0.7%) was of pig origin and 1 of 56 pig worms (1.8%) was of human origin. In China, 13.9% of worms (n = 137) in humans were zoonotic and 0.8% of worms in pigs (n = 121) were anthroponotic [17]. The differences in level of zoonotic transmission between the 2 countries likely represent variations in farming practices, feces disposal, and human-pig contact. Intriguingly, there were 2 worms in pigs from the United Kingdom that clustered with human worms, although one had a pig-like *cox*1 haplotype (H64). These *Ascaris* parasites came from an organic abattoir in Bedfordshire, but the location of the farm(s) where the pigs were reared is not known. It is possible that a human *Ascaris* transmission cycle exists in pigs, an extremely surprising result which warrants further investigation. No examples of hybridization between pig and human worms were found in any location. In contrast, 7.8% of worms in China (n = 258) and 4% in Guatemala (n = 24) were identified as hybrids using 23 microsatellite markers, indicating the potential for exchange of genetic material between pig and human *Ascaris* populations [17, 18]. The fact that we did not find evidence of hybridization might reflect variations in transmission dynamics of *Ascaris* worms in different locations. Alternatively, the 8 markers we used may not provide sufficient resolution to identify hybrids.

As anticipated [4, 13, 18, 20], we found significant genetic differentiation between *Ascaris* populations from different countries. Additional structuring of parasite populations at the village or individual host level was also apparent, particularly in Uganda where the largest sample of worms originated. This may be related to the well-documented overdispersed distribution and clustering of *Ascaris* infections at household level [22, 45]. Genetic substructuring at the individual host level has also been observed in Guatemala and Denmark [4, 14]. The 4 clusters predicted by Bayesian analysis for human worms mainly corresponded to country, although the Ugandan worms also clustered by village. Interestingly, whereas cluster 4 contained mainly worms from Bangladesh, worms from Zanzibar were also found here. Similarly, Zanzibari and some Bangladeshi worms were assigned to cluster 3. This may reflect the long history of and ongoing traffic between Zanzibar and the Indian subcontinent.

Because of greater sampling, our study provides a better picture of the genetic diversity of *Ascaris* in Africa. Since the evolution of early hominids is thought to have taken place here [23], it is possible that there has been a long-standing association between humans and ascarids on this continent and that humans migrating from Africa facilitated the spread of a proto-*Ascaris*. The oldest ascarid eggs discovered so far were found in France [46]. However, Africa is poorly represented in the archeological record and further historical phylogeographical studies are required to determine where *Ascaris* originated. It is clear that, ascariasis has been intimately intertwined with humans for thousands of years, as recently demonstrated by infections discovered within the body of King Richard III [47].

The older evolutionary origin of *Ascaris* in human and pig hosts is contested. It is unclear whether *A. lumbricoides* and *A. suum* are separate species derived from a common ancestor, multiple host colonization events took place after geographical subdivision of host populations [18], or *A. lumbricoides* and *A. suum* are really a single species with regional variants [8]. This discussion depends on a consistent definition of “species.” Defining species as “groups of actually or potentially interbreeding natural populations which are reproductively isolated from other such groups” [48], we suggest that *A. lumbricoides* and *A. suum* are 2 separate species, since there are high levels of genetic differentiation between pigs and human worms in sympatric areas. Although hybrids between pig-derived and human-derived worms have been identified, these are rare and likely to have reduced fitness, otherwise population structuring between the hosts would not be observed [11]. Regarding host switching, our results are less conclusive. Like Anderson et al but in contrast to Criscione et al, we find that, in broad terms, differentiation between worms was based primarily on host affiliation, with further differentiation based on geographical location [13]. This could indicate a single historical host switch of *Ascaris* populations infecting pigs or humans. However, the mitochondrial data and the fact that no single diagnostic marker has been identified to distinguish between *A. lumbricoides* and *A. suum* favor an alternative model of multiple host switches over time, with subsequent merging of worm populations in the 2 hosts [18]. This explanation may be more consistent with the history of pig domestication across Eurasia [49].

The current mainstay of control programs against ascariasis in humans is preventive chemotherapy, involving periodic treatment of preschool and school-aged children in *Ascaris*-endemic communities with anthelmintic drugs, and there are real prospects for local elimination of disease by treatment scale-up [50]. In areas where it is common for households to keep pigs and/or where pig manure is used as a fertilizer, however, the progress toward elimination may be hampered if there is significant cross-over of transmission. Thus, with forthcoming scale up of preventive chemotherapy, it may be that sources of zoonotic transmission will become ever more important, and, if so, they should be taken into account by mathematical models currently being developed to explore the stability and dynamics of end points of control.

In conclusion, our results provide novel insights into the transmission dynamics and speciation of *Ascaris* from humans and pigs. Although *A. lumbricoides* and *A. suum* appear to be 2 separate species over most of their range, the process of speciation is not so far advanced that they are entirely host specific or unable to exchange genetic information, and cross-transmission between pig and human hosts takes place across the globe. The level of cross-transmission is likely to depend on local farming and hygiene practices. However, it is possible that we underestimated the zoonotic potential of *A. suum,* as we only analyzed adult worms (ie, *Ascaris* that completed hepato-tracheal migration and established in the intestine). It is probable that *A. suum* commonly undergoes visceral larvae migration in humans, but because of host preferences most larvae are expelled on return to the intestine. Zoonotic and anthroponotic transmission could lead to greater morbidity because hosts are less well-adapted to the parasite. In addition, potential exchange of genetic information between *A. lumbricoides* and *A. suum* could allow the spread of drug-resistance and virulence genes between parasite populations in different hosts, although it is also possible that if humans are treated but pigs are not, the untreated parasite population will act as refugia, diluting the alleles linked to resistance.

## Supplementary Data

Supplementary materials are available at *The Journal of Infectious Diseases* online (http://jid.oxfordjournals.org/). Supplementary materials consist of data provided by the author that are published to benefit the reader. The posted materials are not copyedited. The contents of all supplementary data are the sole responsibility of the authors. Questions or messages regarding errors should be addressed to the author.

Supplementary Data
