# Supplementary material for: Molecular Epidemiology of Ascariasis: A Global Perspective on the Transmission Dynamics of Ascaris in People and Pigs
Source: J Infect Dis. 2014 Mar 31;210(6):932–41. doi: 10.1093/infdis/jiu193 (PMC4136802; doi:10.1093/infdis/jiu193)
Supplement: Supplementary Data [file supp_210_6_932__index.html]

Molecular Epidemiology of Ascariasis: A Global Perspective on the Transmission Dynamics of Ascaris in People and Pigs — Molecular Epidemiology of Ascariasis: A Global Perspective on the Transmission Dynamics of Ascaris in People and Pigs — Supplementary Data 

# Molecular Epidemiology of Ascariasis: A Global Perspective on the Transmission Dynamics of *Ascaris* in People and Pigs

## Supplementary Data

Supplementary Data

**Files in this Data Supplement:**

- Supplementary Data - Doc file
- Supplementary Table 1 - docx file
- Supplementary Table 2 - docx file
