# Supplementary material for: Molecular Epidemiology of Ascariasis: A Global Perspective on the Transmission Dynamics of Ascaris in People and Pigs
Source: J Infect Dis. 2014 Mar 31;210(6):932–41. doi: 10.1093/infdis/jiu193 (PMC4136802; doi:10.1093/infdis/jiu193)

**Supporting Information Legends**

**Table S1.** Microsatellite allelic diversity by marker, host and country.

**Table S2.** Individual *Ascaris* worms identified as potential cross infections or hybrids.

**Figure S1.** Maximum likelihood tree displaying the phylogenetic relationships between *cox*1 haplotypes based on the Hasegawa-Kishina-Yano model. Haplotypes from Cavallero et al [2013] (CavHap) are included for comparison as well as *cox*1 sequences from *Baylisascaris transfuga*, *B*. *ailuri*, *B*. *schroederi*, *B*. *columnaris*, *Toxocara cati*, *T*. *canis* and *Toxascaris leonina* to act as outgroups. Asterisks indicate branches for which bootstrap support was over 75% (1000 bootstraps). Squares – haplotypes found in worms from human hosts; circles – haplotypes found in worms from pig hosts; red shapes –worms from Africa; green shapes – worms from Asia; orange shapes –worms from Latin America; blue shapes – worms from Europe. A, B and C refer to the three clusters identified in Figure 1.


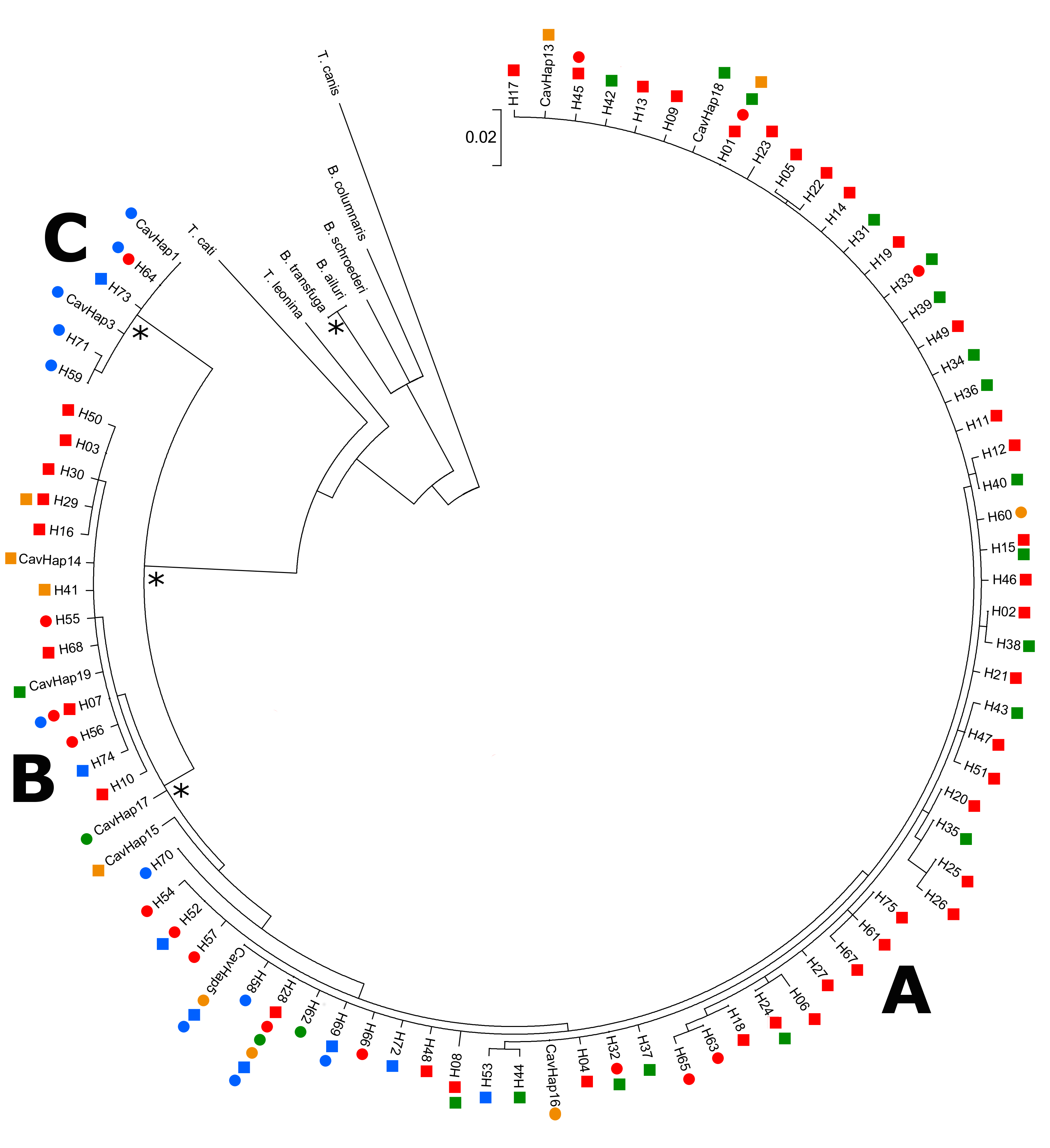

Supplement: Supplementary Data [file supp_jiu193_jiu193supp.doc]
