# Supplementary material for: Molecular Epidemiology of Ascariasis: A Global Perspective on the Transmission Dynamics of Ascaris in People and Pigs
Source: J Infect Dis. 2014 Mar 31;210(6):932–41. doi: 10.1093/infdis/jiu193 (PMC4136802; doi:10.1093/infdis/jiu193)
Supplement: Supplementary Data [file supp_jiu193_jiu193supp_table1.docx]

**Table S1.** Microsatellite allelic diversity by marker, host and country

| Locus |  | PUK^a^  (*N*=41)^b^ | HUK  (*N*=11) | PDK  (*N*=31) | HUG  (*N*=143) | PUG  (*N*=56) | HZZ  (*N*=53) | PTZ  (*N*=36) | HBA  (*N*=37) | ALL  (*N*=408) |
| --- | --- | --- | --- | --- | --- | --- | --- | --- | --- | --- |
| ALTN04 | N_A_ (N_P_)^c^ | 8 (0) | 10 (0) | 12 (0) | 13 (0) | 11 (0) | 16 (1) | 12 (0) | 12 (0) | 24 |
|  | R_A_ | 4.41 | 10.00 | 7.94 | 4.85 | 6.73 | 6.97 | 7.76 | 7.35 | 8.41 |
|  | H_O_ | 0.487 | 0.727 | 0.774 | 0.416 | 0.564 | 0.750 | 0.543 | 0.647 | 0.554 |
|  | H_E_ | 0.458 | 0.826 | 0.791 | 0.432 | 0.785 | 0.723 | 0.830 | 0.725 | 0.714 |
|  | F_IS_ | -0.049 | 0.167 | 0.038 | 0.042 | 0.290 | -0.025 | 0.359 | 0.123 | 0.2247 |
| ALAC09 | N_A_ (N_P_) | 10 (0) | 6 (0) | 7 (0) | 12 (0) | 9 (0) | 9 (0) | 9 (0) | 12 (1) | 17 |
|  | R_A_ | 7.280 | 6.000 | 4.956 | 7.240 | 5.139 | 6.338 | 5.293 | 7.673 | 8.21 |
|  | H_O_ | 0.590 | 0.546 | 0.552 | 0.768 | 0.714 | 0.660 | 0.333 | 0.639 | 0.656 |
|  | H_E_ | 0.797 | 0.736 | 0.576 | 0.827 | 0.698 | 0.804 | 0.541 | 0.753 | 0.844 |
|  | F_IS_ | 0.272 | 0.302 | 0.059 | 0.075 | -0.014 | 0.187 | 0.395 | 0.165 | 0.2245 |
| ALGA48 | N_A_ (N_P_) | 16 (2) | 7 (1) | 11 (0) | 15 (1) | 14 (1) | 14 (2) | 11 (1) | 15 (2) | 31 |
|  | R_A_ | 11.243 | 7.000 | 9.535 | 5.569 | 8.137 | 6.121 | 7.690 | 7.982 | 9.102 |
|  | H_O_ | 0.824 | 0.636 | 0.759 | 0.489 | 0.661 | 0.549 | 0.514 | 0.571 | 0.586 |
|  | H_E_ | 0.891 | 0.740 | 0.892 | 0.748 | 0.822 | 0.737 | 0.831 | 0.813 | 0.852 |
|  | F_IS_ | 0.091 | 0.186 | 0.167 | 0.350 | 0.205 | 0.264 | 0.394 | 0.310 | 0.3135 |
| ALAC07 | N_A_ (N_P_) | 14 (2) | 8 (1) | 11 (1) | 16 (3) | 12 (0) | 16 (1) | 9 (0) | 15 (2) | 31 |
|  | R_A_ | 8.455 | 8.000 | 8.433 | 7.140 | 7.974 | 8.282 | 6.557 | 9.523 | 9.639 |
|  | H_O_ | 0.611 | 0.273 | 0.774 | 0.752 | 0.732 | 0.824 | 0.528 | 0.886 | 0.725 |
|  | H_E_ | 0.850 | 0.802 | 0.820 | 0.809 | 0.832 | 0.835 | 0.802 | 0.858 | 0.863 |
|  | F_IS_ | 0.294 | 0.686 | 0.072 | 0.074 | 0.129 | 0.024 | 0.354 | -0.017 | 0.1611 |
| ALGA31 | N_A_ (N_P_) | 9 (1) | 7 (0) | 10 (1) | 18 (1) | 8 (0) | 13 (1) | 11 (0) | 8 (0) | 24 |
|  | R_A_ | 4.559 | 7.000 | 5.651 | 7.533 | 5.766 | 7.584 | 7.146 | 6.184 | 8.525 |
|  | H_O_ | 0.485 | 0.546 | 0.433 | 0.684 | 0.491 | 0.698 | 0.533 | 0.344 | 0.576 |
|  | H_E_ | 0.589 | 0.570 | 0.637 | 0.808 | 0.712 | 0.753 | 0.651 | 0.739 | 0.790 |
|  | F_IS_ | 0.192 | 0.091 | 0.335 | 0.157 | 0.319 | 0.082 | 0.197 | 0.546 | 0.2719 |
| ALGA15 | N_A_ (N_P_) | 15 (3) | 9 (0) | 10 (0) | 30 (5) | 15 (0) | 21 (5) | 15 (3) | 20 (0) | 48 |
|  | R_A_ | 9.660 | 9.000 | 7.540 | 9.421 | 8.493 | 11.366 | 9.055 | 11.776 | 12.514 |
|  | H_O_ | 0.641 | 0.818 | 0.742 | 0.701 | 0.589 | 0.769 | 0.583 | 0.767 | 0.686 |
|  | H_E_ | 0.881 | 0.860 | 0.778 | 0.872 | 0.815 | 0.900 | 0.858 | 0.901 | 0.920 |
|  | F_IS_ | 0.284 | 0.095 | 0.063 | 0.200 | 0.285 | 0.132 | 0.332 | 0.165 | 0.2553 |
| ALAC08 | N_A_ (N_P_) | 15 (2) | 7 (0) | 9 (0) | 30 (4) | 17 (0) | 22 (3) | 14 (0) | 17 (1) | 42 |
|  | R_A_ | 10.787 | 7.000 | 7.233 | 11.454 | 9.710 | 12.607 | 7.765 | 10.323 | 13.534 |
|  | H_O_ | 0.697 | 0.727 | 0.903 | 0.757 | 0.804 | 0.706 | 0.514 | 0.771 | 0.744 |
|  | H_E_ | 0.900 | 0.831 | 0.840 | 0.903 | 0.876 | 0.911 | 0.726 | 0.827 | 0.939 |
|  | F_IS_ | 0.240 | 0.171 | -0.059 | 0.164 | 0.091 | 0.240 | 0.305 | 0.081 | 0.2093 |
| ALAC32 | N_A_ (N_P_) | 16 (6) | 9 (0) | 11 (0) | 25 (1) | 15 (1) | 22 (3) | 10 (0) | 21 (2) | 40 |
|  | R_A_ | 9.431 | 9.000 | 8.531 | 11.665 | 7.568 | 10.985 | 7.042 | 11.459 | 12.243 |
|  | H_O_ | 0.778 | 0.636 | 0.710 | 0.736 | 0.655 | 0.813 | 0.559 | 0.618 | 0.707 |
|  | H_E_ | 0.859 | 0.835 | 0.859 | 0.913 | 0.773 | 0.879 | 0.771 | 0.896 | 0.920 |
|  | F_IS_ | 0.109 | 0.282 | 0.190 | 0.198 | 0.162 | 0.086 | 0.289 | 0.324 | 0.2359 |
| ALL | Mean N_A_  (SD)^d^ | 12.88  (3.31) | 7.88  (1.36) | 10.13  (1.55) | 19.88  (7.40) | 12.63  (3.16) | 16.63  (4.72) | 11.38  (2.20) | 15.00  (4.34) | 32.25  (10.74) |
|  | Mean R_A_  (SD) | 8.23  (2.62) | 7.88  (1.36) | 7.48  (1.52) | 8.11  (2.53) | 7.44  (1.50) | 8.78  (2.51) | 7.29  (1.09) | 9.03  (2.04) | 10.27  (2.14) |
|  | Mean H_O_  (SD) | 0.639  (0.124) | 0.614  (0.167) | 0.706  (0.147) | 0.663  (0.135) | 0.651  (0.101) | 0.721  (0.089) | 0.514  (0.076) | 0.655  (0.162) | 0.654  (0.073) |
|  | Mean H_E_  (SD) | 0.778 (0.164) | 0.775  (0.094) | 0.774  (0.167) | 0.789  (0.154) | 0.789  (0.060) | 0.818  (0.075) | 0.751  (0.108) | 0.814  (0.069) | 0.855  (0.076) |
|  | Overall F_IS_ | 0.193 | 0.253 | 0.105 | 0.164 | 0.184 | 0.126 | 0.330 | 0.209 | 0.236 |

^a^ H = worms from human hosts; P = worms from pig hosts; UG = Uganda; TZ =Tanzania; ZZ =Zanzibar; KE =Kenya; ZA = Zambia; BA = Bangladesh; NP = Nepal; PH = Philippines; GT =Guatemala; DK = Denmark; UK= United Kingdom. ^b^ *N* = number of samples; ^c^ N_A_ = number of alleles; N_P_= number of private alleles; R_A_ = allelic richness; H_O_ = observed heterozygosity; H_E_ = expected heterozygosity; F_IS_ = inbreeding coefficient. ^d^ SD= standard deviation.
