# Supplementary material for: Molecular Epidemiology of Ascariasis: A Global Perspective on the Transmission Dynamics of Ascaris in People and Pigs
Source: J Infect Dis. 2014 Mar 31;210(6):932–41. doi: 10.1093/infdis/jiu193 (PMC4136802; doi:10.1093/infdis/jiu193)
Supplement: Supplementary Data [file supp_jiu193_jiu193supp_table2.docx]

**Table S2.** Individual *Ascaris* worms identified as potential cross infections or hybrids^a^

| Host | Country | *Ascaris* ID | STRUCTURE  *Q*-human (90%PI)  *Q*-pig (90%PI) | BAPS  *Q*-human  *Q*-pi (*p* value) | NewHybrids  pp-category  (%) | Classification | Conclusion |
| --- | --- | --- | --- | --- | --- | --- | --- |
| Human | UK | HUK1.1 | 0.704 (0.000,1.000)  0.296 (0.000,1.000) | 0.00  1.00 (*p*=1.00) | 80.6 human | human (NH)  pig (B), hybrid (S) | undetermined |
|  | Denmark | HDK1.2 | 0.702 (0.000,1.000)  0.298 (0.000,1.000) | 0.00  1.00 (*p*=1.00) | 80.6 human | human (NH)  pig (B), hybrid (S) | undetermined |
|  | Uganda | HAB5.1 | 0.995 (0.975,1.000)  0.005 (0.000,0.025) | 1.00 (*p*=1.00)  0.00 | 99.4 pig | human (S, B)  pig (NH) | undetermined |
|  |  | MUS1.2 | 0.568 (0.000,1.000)  0.432 (0.000,1.000) | 1.00 (*p*=1.00)  0.00 | 55.9 pig  30.6 hybrid | pig (B, NH)  hybrid (S) | undetermined |
|  |  | **MUS5.10** | **0.011 (0.000,0.059)**  **0.989 (0.941,1.000)** | **0.00**  **1.00 (*p*=1.00)** | **99.3 pig** | **pig (S, B, NH)** | **pig worm** |
|  |  | HUG14.1 | 0.996 (0.983,1.000)  0.004 (0.000,0.017) | 1.00 (*p*=1.00)  0.00 | 99.4 pig | human (S, B)  pig (NH) | undetermined |
|  | Zanzibar | G8 | 0.990 (0.948,1.000)  0.010 (0.000,0.052) | 1.00 (*p*=1.00)  0.00 | 99.8 pig | human (B, S)  pig (NH) | undetermined |
| Pig | UK | PUK2.6 | 0.704 (0.194,1.000)  0.296 (0.000,0.806) | 1.00 (*p*=1.00)  0.00 | 77.2 human  21.0 hybrid | human (B, NH)  hybrid (S) | undetermined  (human) |
|  |  | PUK2.9 | 0.577 (0.000,1.000)  0.423 (0.000,1.000) | 0.00  1.00 (*p*=1.00) | 61.8 human  26.4 pig | human (NH)  pig (B), hybrid (S) | undetermined |
|  |  | **PUK3.2** | **0.899 (0.000,1.000)**  **0.101 (0.000,1.000)** | **1.00 (*p*=1.00)**  **0.00** | **93.9 human** | **human (S, B, NH)** | **human worm** |
|  |  | PUK4.18 | 0.478 (0.000,1.000)  0.522 (0.000,1.000) | 0.00  1.00 (*p*=1.00) | 52.7 human  44.0 pig | human (NH)  pig (B), hybrid (S) | undetermined |
|  |  | PUK4.21 | 0.215 (0.000,1.000)  0.785 (0.000,1.000) | 0.00  1.00 (*p*=1.00) | 70.5 pig  23.3 human | pig (NH, B)  hybrid (S) | undetermined  (pig) |
|  |  | PUK4.7 | 0.409 (0.000,1.000)  0.591 (0.000,1.000) | 1.00 (*p*=1.00)  0.00 | 50.0 pig  38.6 human | human (B), hybrid (S)  undetermined (NH) | undetermined |
|  |  | **PUK4.9** | **0.959 (0.741,1.000)**  **0.041 (0.000,0.259)** | **1.00 (*p*=1.00)**  **0.00** | **96.9 human** | **human (S, B, NH)** | **human worm** |
|  | Uganda | PUG1.1 | 0.574 (0.000,1.000)  0.476 (0.000,1.000) | 0.00  1.00 (*p*=1.00) | 45.7 human  34.0 pig | pig (B), hybrid (S)  undetermined (NH) | undetermined |
|  |  | **PUG3.2** | **0.997 (0.985,1.000)**  **0.003 (0.000,0.015)** | **1.00 (p=1.00)**  **0.00** | **99.8 human** | **human (S, B, NH)** | **human worm** |
|  |  | PUG7.1 | 0.680 (0.000,1.000)  0.320 (0.000,1.000) | 0.00  1.00 (*p*=1.00) | 60.7 pig  23.2 hybrid | pig (B, NH)  hybrid (S) | undetermined |
|  |  | PUG7.3 | 0.344 (0.000,1.000)  0.656 (0.000,1.000) | 0.00  1.00 (*p*=1.00) | 65.1 pig  19.0 human | pig (B, NH)  hybrid (S) | undetermined |
|  | Tanzania | PTZ1.1 | 0.214 (0.000,0.984)  0.786 (0.016,1.000) | 0.00  1.00 (*p*=1.00) | 62.4 pig  20.7 hybrid | pig (B, NH)  hybrid (S) | undetermined  (pig) |
|  |  | PTZ1.2 | 0.680 (0.000,1.000)  0.320 (0.000,1.000) | 1.00 (*p*=1.00)  0.00 | 77.0 human  14.9 hybrid | human (B, NH)  hybrid (S) | undetermined |
|  |  | PTZ7.1 | 0.780 (0.289,1.000)  0.220 (0.000,0.711) | 1.00 (*p*=1.00)  0.00 | 73.3 human  24.2 hybrid | human (B, NH)  hybrid (S) | undetermined  (human) |

a For simplicity, the 11 worms from humans in UK and Denmark which were unequivocally identified as pig worms by all three programmes have not been included.
